# Expanding the Immunologic and Neuronal Landscape of IgE‐Mediated Anaphylaxis

**DOI:** 10.1111/imr.70078

**Published:** 2025-12-02

**Authors:** Ruchi Biswas, Jamie Moore Fried, Maria A. Curotto de Lafaille

**Affiliations:** ^1^ Rutgers New Jersey Medical School Newark New Jersey USA; ^2^ Serena and John Liew Division of Pediatric Allergy and Immunology, Department of Pediatrics, Elliot and Roslyn Jaffe Food Allergy Institute Icahn School of Medicine at Mount Sinai (ISMMS) New York New York USA; ^3^ Kravis Children's Hospital Icahn School of Medicine at Mount Sinai (ISMMS) New York New York USA; ^4^ Department of Immunology and Immunotherapy Marc and Jennifer Lipschutz Precision Immunology Institute, Icahn School of Medicine at Mount Sinai (ISMMS) New York New York USA

**Keywords:** anaphylaxis, augmenting factors of anaphylaxis, food allergy, IgE, mast cell, neuro‐immune interactions, neuronal signaling

## Abstract

Anaphylaxis is a life‐threatening immunoglobulin E (IgE)‐mediated type I hypersensitivity reaction with rising prevalence and burden. It involves mast cell degranulation upon cross‐linking of antigen on mast cell‐bound IgE. Mechanisms of IgE‐mediated anaphylaxis remain incompletely understood, particularly the induction of systemic symptoms (hypothermia, hypotension). We consider two hypotheses driving anaphylaxis. In the first case, circulating antigen reaches mast cells systemically, causing widespread degranulation and downstream effects. In a second scenario, a subset of mast cells “view” antigen, initiating local activation and extensive neuronal signaling to activate distal mast cells and trigger tissue responses. Support for systemic antigen is evident in food allergy, with allergenicity determined by antigen stability in the face of digestion, and antigen translocation across the gut epithelium and within circulation, possibly through chylomicrons. Emerging research implicates neuronal signaling in modulating systemic responses, with mast cells communicating bidirectionally with neurons via released mediators. These interactions lower activation thresholds, amplify inflammation, and engage key downstream receptors and pathways. In vivo models demonstrate such mast cell neuromodulation underlying systemic manifestations of IgE‐mediated anaphylaxis, including pruritus and hypothermia. The evidence suggests that both scenarios are likely at play in anaphylaxis, warranting further investigation.

## 1 Introduction

### 1.1 Definition

Anaphylaxis is broadly defined to be a serious allergic hypersensitivity reaction that can rapidly progress and may result in death [1]. In recent years, experts have developed standardized definitions of anaphylaxis that capture a spectrum of clinical scenarios. First, in the event of a known allergen exposure, acute onset (within minutes to hours) of respiratory or cardiovascular compromise alone may constitute a diagnosis of anaphylaxis. Second, in cases with a likely or known allergen exposure, a diagnosis may be made when two or more body systems are affected—such as the skin, mucosa, respiratory tract, cardiovascular system, or gastrointestinal (GI) tract. When there is no known allergic trigger, anaphylaxis may be likely if one develops sudden onset of skin or mucosal symptoms together with respiratory or cardiovascular involvement [1]. Cutaneous findings can include urticaria, flushing, erythema, facial swelling, or in infants, mottled skin. Mucosal symptoms may involve swelling of the lips, tongue, or oropharynx, as well as throat tightness or difficulty swallowing. In infants, repetitive lip‐licking can be an early mucosal sign. Respiratory manifestations can present as wheezing, a cough (often a repetitive staccato‐type cough), shortness of breath, hypoxemia, voice changes, or stridor. Cardiovascular involvement may include syncope, dizziness, altered mental status or hypotension, and tachycardia in the case of infants. Severe abdominal pain, repetitive vomiting, and diarrhea comprise possible gastrointestinal manifestations of anaphylaxis [1, 2, 3, 4]. Although not required for diagnosis, acute elevations in basal serum tryptase (BST), defined as 20% plus 2 ng/mL above baseline tryptase levels, support the diagnosis of anaphylaxis and can help identify underlying mast cell disorders [5, 6, 7].

Anaphylaxis may be further categorized based on clinical course. Biphasic anaphylaxis can be determined when there is a recurrence of symptoms of anaphylaxis within 48 h after a period of full resolution, without re‐exposure to the allergen. Protracted anaphylaxis is considered to occur when symptoms persist for 4 h or longer, while refractory anaphylaxis continues despite appropriate treatment and carries an increased risk of fatality [4, 8, 9, 10, 11, 12, 13].

### 1.2 Epidemiology

Anaphylaxis has an estimated lifetime prevalence of 0.5% to 2% in the US population, with an estimated 2.3‐fold increase in emergency department (ED) visits, due to anaphylaxis, between 2008 and 2016, and a 4.2% per‐year increase in pediatric ED visit incidence from 2016 to 2022 [14, 15, 16]. The prevalence of food allergy in adults was 10.8%, with 51.1% of food‐allergic individuals experiencing an allergic reaction, and 38.3% having experienced at least one ED visit due to their allergy [17]. The most common cause of pediatric anaphylaxis includes accidental ingestion of food allergens such as peanuts, tree nuts, shellfish, eggs, and cow's milk [2, 18]. Children and adolescents are the most prevalent group implicated in overall cases of food‐induced anaphylaxis, with pediatric cases having increased by 3.2‐fold between 2008 and 2016 [14, 15, 19]. Adult cases of anaphylaxis have more varied etiologies, including drug administration, insect stings, latex, and idiopathic causes [2]. Female sex and advanced age are recognized modifiers of anaphylaxis severity [20]. Additionally, studies corroborate the significant impact of food allergy and previous history of anaphylaxis on patient quality of life [21]. Death rates due to anaphylaxis are between 0.47 to 0.69 per million persons annually [22, 23, 24, 25].

Higher rates of sensitization to foods and incidents of anaphylaxis, requiring ED visits, as well as greater severity of symptoms, have been reported in Black and Hispanic populations [17, 26, 27, 28]. Urban populations face greater allergen exposures that contribute to atopic disease, underscoring the need to better understand sensitization and anaphylaxis—a condition of rising prevalence, morbidity, and, often underestimated, economic burden [15, 17, 29, 30]. Studies show that low‐income children incur 2.5 times the cost of emergency and specialty visits compared to their higher‐income counterparts, outlining a particularly vulnerable population [27, 31]. Additional at‐risk groups include patients with atopic disease, mast cell disorders, and multiple food allergies. Cofactors—that reduce the allergic thresholds and risk of anaphylaxis—include medication use (nonsteroidal anti‐inflammatory drugs, angiotensin inhibitors, beta‐blockers), alcohol use, exercise, temperature, and history of concurrent illness [2, 20, 32]. In all, there is a need for a better understanding of anaphylaxis to reduce the overall disease prevalence and mortality, especially among predisposed and disproportionately affected groups.

### 1.3 Clinical Management

Treatment of anaphylaxis is focused on prevention and reversal of cardiovascular or respiratory collapse. The mainstay and only approved treatment, epinephrine—an α‐ and β‐adrenergic receptor agonist—is administered intramuscularly to therapeutically vasoconstrict, bronchodilate, and reduce mucosal edema [4, 33]. Prompt recognition and immediate administration of epinephrine are critical, as delayed use is associated with worse outcomes [4].

Additional supportive care measures may be indicated as clinically relevant. Aggressive intravenous fluid resuscitation should be employed when hypotension or signs of vital organ hypoperfusion are suspected [4, 34]. Importantly, individuals suspected of having anaphylaxis should not ambulate and should remain supine, as hemodynamic changes associated with ambulation may result in empty ventricle syndrome with catastrophic results [35, 36]. Supplemental oxygen may be used in cases of hypoxemia, and albuterol, a β‐2‐adrenergic receptor agonist, may be coadministered to mitigate bronchospasm [33]. In cases of refractory anaphylaxis, an epinephrine drip may be indicated [34, 36, 37, 38]. If there is respiratory or cardiac failure, intubation or advanced mechanical support may be required [34, 36, 37, 38].

Of note, antihistamines do not reverse airway or cardiovascular compromise and thus are not recommended for the acute management of anaphylaxis. Antihistamines may be used as an adjunctive treatment after clinical stabilization [25, 38, 39, 40, 41]. A cohort study in Canada demonstrated that prehospital use of antihistamines reduced the likelihood of receiving multiple doses of epinephrine [42]. A combination of high‐dose H1 (histamine type 1 receptor) and H2 (histamine type 2 receptor) antihistamines is recommended to reduce symptoms in patients with recurrent idiopathic anaphylaxis and systemic mastocytosis, although there is not enough robust evidence that antihistamines prevent anaphylaxis [43, 44, 45]. While steroids have been administered as part of an anaphylaxis management, with the thought that they may help prevent biphasic reactions, meta‐analyses failed to demonstrate a protective benefit, and their use is not recommended [4, 38, 46].

The only reliable strategy to prevent anaphylaxis is through the strict avoidance of triggers [4, 38]. However, new agents show promise in reducing the risk of developing anaphylaxis. Omalizumab, an anti‐immunoglobulin (Ig)E monoclonal antibody, has been shown to increase the threshold for hypersensitivity reactions by preventing IgE cross‐linking on mast cells (MCs), leading to downregulation of surface IgE receptors, demonstrating utility in idiopathic anaphylaxis, hereditary alpha tryptasemia (HαT), food allergy, and further improving safety in desensitization via oral immunotherapy [47, 48, 49, 50, 51]. Dupilumab, an interleukin (IL)‐4 receptor antagonist, inhibits the IL‐4 and IL‐13 signaling cascade, both of which are important in IgE class switching and production of IgE. Although mechanistically, dupilumab was thought to be a promising candidate for reducing the incidence of anaphylaxis, a phase II single‐arm clinical trial in peanut‐allergic children found that dupilumab did not increase the reaction threshold or improve the likelihood of achieving desensitization to peanut [52]. In a case study by Otani et al., treatment with dupilumab was effective in preventing recurrent anaphylaxis in a patient with severe asthma; however, further studies are required to confirm the potential utility of this biologic in anaphylaxis prevention [53, 54]. Lastly, MC cytoreduction has limited but emerging data suggesting potential benefit in decreasing MC burden and thereby reducing mediator releasein patients with systemic mastocytosis, though its effect on anaphylaxis risk remains unclear [55, 56].

Additional studies propose to therapeutically target a variety of pathways and kinases implicated in MC activation and degranulation, including mitogen‐activated protein kinases (MAPK), phosphoinositide‐specific phospholipase C (PLCγ), Bruton Kinase (BTK), and phosphoinositide 3‐kinase (PI3K) pathways [54, 57, 58, 59]. Indeed, in a phase II clinical trial, the BTK inhibitor acalabrutinib did show efficacy in increasing the reaction threshold among patients with peanut allergy [60].

Preventive strategies act on upstream mediators, such as IgE, to reduce the risk of anaphylactic events. However, once anaphylaxis has occurred, these agents have no proven role in the acute setting. Anti‐IgE therapy, such as omalizumab, does not provide any acute benefit once the reaction is underway [48]. This distinction underscores an important mechanistic concept: IgE and histamine are upstream mediators in the cascade that leads to anaphylaxis, whereas epinephrine acts downstream, reversing the severe pathophysiologic processes at end organs. The absence of preventive therapies targeting the downstream pathways of anaphylaxis further highlights the need to investigate its underlying mechanisms.

### 1.4 Etiology

Type I hypersensitivity reactions are characterized by IgE‐mediated activation and degranulation of MCs and basophils following antigen‐induced crosslinking [1, 3, 4, 61, 62]. These events underlie immediate hypersensitivity reactions, which are responsible for food, venom, and drug allergies [3, 4, 63, 64]. Such reactions can result in classic anaphylaxis. In this review, we focus on MCs in anaphylaxis.

Sensitization from prior exposure results in the production of IgE antibodies, which bind to the Fc epsilon receptor (FcεRI) expressed on the surface of MCs and basophils. Upon subsequent exposure, antigen crosslinks to surface‐bound IgE, triggering rapid degranulation of MCs, resulting in the release of preformed inflammatory mediators (e.g., histamine, proteases, carboxypeptidase A, heparin/proteoglycans, tumor necrosis factor (TNF)‐α) within seconds to minutes. This is followed by the de novo synthesis of lipid mediators (e.g., leukotrienes, prostaglandin D2), platelet‐activating factor (PAF), neuropeptides, and multiple cytokines and chemokines (IL‐1, IL‐4, IL‐5, IL‐6, IL‐13, TNF‐α) [36, 54, 62, 65, 66].

The relative role of each pre‐formed and synthesized mediator in potentiating anaphylaxis is not fully delineated. Histamine promotes vasodilation, increased vascular permeability, tachycardia, and glandular secretion [62, 67]. Prostaglandin D2 acts as a bronchoconstrictor, vasoconstrictor of pulmonary and cardiac vessels, and peripheral vasodilator [62]. Leukotrienes and PAF further potentiate bronchoconstriction and increased vascular permeability [62, 67]. TNF‐α functions both as an early preformed mediator, activating neutrophils and recruiting effector cells, and as a late‐phase mediator in biphasic or protracted anaphylactic responses [62, 67].

Elevated levels of total and specific IgE (sIgE) are present in allergic individuals, with allergen sIgE serving as a key diagnostic and predictive marker of true allergy when interpreted alongside clinical history and skin testing [4, 68, 69]. However, IgE levels do not reliably predict the severity of a reaction or the propensity to develop anaphylaxis. Indeed, individuals with relatively low sIgE levels may still experience severe reactions, suggesting that other factors contribute to this likelihood of developing anaphylaxis [70, 71, 72].

MCs, of myeloid lineage, are the principal population of granulocyte effector cells implicated in anaphylactic reactions. This cell population has a high number of high‐affinity FcεRIs and is highly localized within the mucosal surfaces and connective tissue of the intestines, skin, and respiratory tract/lungs [54, 70, 71, 73, 74, 75, 76, 77]. MCs' localization near blood vessels, smooth muscle, and mucous membranes underscores the broad range of tissues affected upon activation [70, 71, 73, 74, 76]. Their perivascular distribution is of specific interest, potentially directly contributing to changes in vascular permeability and tone seen in anaphylaxis [74].

MCs have mixed embryonic origins, arising from both yolk sac and hematopoietic stem cell‐derived progenitors, and mature within tissues of nearly all organs, where they become part of the innate immune cell population [78, 79, 80, 81, 82]. Based on histochemical and functional characteristics, MCs are broadly categorized into two major subtypes: mucosal mast cells (MMCs) and connective tissue mast cells (CTMCs). MMCs, derived primarily from hematopoietic progenitors, are abundant within the mucosa of the gut and lungs. In contrast, CTMCs originate from yolk sac precursors and are thought to undergo renewal from bone marrow‐derived progenitors and reside predominantly within the skin and peritoneal cavity [78, 82, 83]. MC populations display distinct receptor expression profiles and granule content depending on their tissue localization. MMCs are enriched for tryptase, the biomarker for anaphylaxis, while CTMCs are histamine‐skewed [73, 84].

Although tryptase elevation is characteristic of anaphylaxis, it is less consistently observed in food‐induced reactions than in drug‐ or venom‐induced anaphylaxis. While MMCs could reasonably be presumed to first encounter allergenic material within the mucosa of the GI tract and degranulate in response, tryptase is often not significantly elevated in food‐mediated anaphylaxis, and the basis for this discrepancy is unclear [4, 85, 86, 87]. MCs' proximity to neurovascular bundles, allowing for bidirectional signaling via neuro‐immune synapses, may underlie this phenomenon, but it is still an active area of investigation [66, 73, 74, 88, 89, 90].

### 1.5 Genetics and Mast Cell Disorders

Some clues about the mechanisms underlying anaphylaxis come from genetic and acquired conditions associated with increased susceptibility, including HαT and mastocytosis, as well as single‐nucleotide polymorphisms (SNPs) in the IL‐4 receptor (IL‐4R).

HαT is a condition with an autosomal dominant inheritance pattern wherein there is an increase in the α‐tryptase‐encoding tryptase α/β‐1 (TPSAB1) gene copy number, resulting in elevated BST levels (typically > 8 ng/mL) [91, 92]. Studies estimate the prevalence of HαT to be approximately 6%. Severe anaphylaxis, commonly idiopathic or toxin‐induced, occurs in about 14% to 28% of these affected individuals, making HαT one of the few heritable diseases predisposing to anaphylaxis [93, 94, 95]. In vitro, activated tryptases have been shown to stimulate neuronal activity, modulate smooth muscle contraction, activate key downstream pathways (via tissue matrix metalloproteinases and vasoactive intestinal peptides), promote vascular permeability, and even further perpetuate MC degranulation in a proactive signaling cascade—actions that promote, or are evident in, anaphylaxis [91]. However, the elevated levels of precursor, monomeric, tryptases in HαT are inert. Only once enzymatically active as tetramers, through pathways not yet fully elucidated and not clearly established in HαT, are they able to produce this disease manifestation [91, 93, 95, 96].

Mastocytosis is defined by an increase in MC burden, tissue infiltration, and release of mediators inducing systemic anaphylaxis, with manifestations ranging from cutaneous involvement to severe organ infiltration. Exceedingly rare, it has an estimated prevalence of 0.01%. It is characterized by an activating mutation in the C‐KIT gene (D816V), encoding a key tyrosine kinase receptor on MCs. Studies postulate that this mutation impacts MC activity, growth, and apoptosis, resulting in unregulated expansion of this cell population through constitutive activation of tyrosine kinase and, subsequently, the STAT5–PI3K–Akt signaling cascade [55, 97, 98]. Harir et al. [99] show that activation of this pathway in vitro and in vivo results in the growth of human hematopoietic progenitor cells, and ultimately the neoplastic MC proliferation seen in systemic mastocytosis.

Other hypotheses suggest an additional role for the KIT receptor ligand, stem cell factor, where overexpression may result in increased MC activation and subsequently hyperplasia. However, studies have shown mixed results in establishing this correlation [100, 101]. Interestingly, Lyons [91] reported that HαT was significantly more prevalent in patients with mastocytosis (2.5‐fold higher) and idiopathic anaphylaxis (3‐fold higher). With this, clinical guidelines recommend that patients with unexplained anaphylaxis or a history of severe reactions obtain baseline tryptase serum levels [4].

A genetic predisposition to anaphylaxis has been linked to SNPs in the IL‐4Rα gene. The IL‐4 and IL‐13 signaling pathways are integral to allergic responses; IL‐4 and IL‐13 have key receptor targets, notably IL‐4R, activating the STAT6 and PI3K pathways. IL‐4Rα, a subunit of IL‐4R, contains multiple positive and negative regulatory domains that modulate signaling. Activation of IL‐4Rα is necessary for this downstream signaling, regulating IL‐4/IL‐13 responses and ultimately pro‐allergic type 2 inflammation and MC hyperplasia. This is demonstrated in clinical studies examining radioallergoabsorbant (RAST) assays in patients with SNPs; atopic patients exhibited a higher incidence of activating 1652A/G IL‐4Rα SNPs, and had significantly higher frequencies of positive RAST testing [102]. Additionally, Mathias et al. [103] showed that activating mutations in IL‐4Rα in murine models increase gastrointestinal permeability to allergens, IgE, and MC burden, thereby increasing the risk for food‐induced anaphylaxis. Interestingly, Sledd et al. [104] suggested that allergic susceptibility arises not only from gain‐of‐function mutations, but also from loss‐of‐function mutations with impaired IL‐4Rα/PI3K signaling attenuating IL‐4 driven CD4^+^ T‐cell proliferation, reducing T cell tolerance, enhancing IgE production, and predisposing to allergic asthma and anaphylaxis. In a murine model, Sledd and colleagues [104] showed that disruption of an IL‐4Rα signaling domain (Y497 motif of IL‐4Rα in humans, Y500 in mice), activates PI3K, increases the extent, rate, and acceleration of systemic anaphylactic symptoms—hypothermia and hypotension—following oral exposure to allergen. It was further found that IL4R‐PI3K signaling in endothelial cells negatively regulates the increased vascular permeability response to histamine [104]. The exact mechanism of action of IL‐4Rα on allergic and anaphylactic predisposition is thus not fully understood and is likely multifactorial; nonetheless, this illustrates the complexity of effects that IL‐4Rα gene polymorphisms have on allergic phenotypes.

In all, these conditions support that increases in MCs, their mediators, the IL‐4 signaling cascade, and the formation of allergic IgE antibodies collectively promote anaphylaxis.

### 1.6 Mechanistic Hypotheses

Although contributors to IgE‐mediated anaphylaxis are known, its deeper immunologic mechanisms and relative importance of mediators remain unclear. Our current understanding of classic IgE‐mediated anaphylaxis suggests that MCs must be exposed to allergen to mediate systemic anaphylaxis [75, 105, 106]. The limited number of IgE‐producing cells and the short half‐life of circulating IgE, together with the presence of circulating allergen‐specific IgG, indicate that IgE does not form circulating immune complexes with allergen [107, 108, 109]. Thus, allergen needs to directly reach MCs to induce IgE‐crosslinking‐mediated activation. However, direct allergen‐mediated MC activation may not fully account for systemic degranulation and widespread, downstream effects.

Systemic and oral mechanisms of anaphylaxis differ, evidenced in distinctions in MC populations and localizations dictating their ability to sample their surroundings and degranulate accordingly. Local degranulation of MCs occurs following antigen cross‐linking of surface‐bound IgE. While several studies have examined this process in the GI tract, the rapid onset of systemic effects after ingestion—prior to the allergen reaching the small intestine—suggests that MC degranulation in more proximal, local sites, such as the oropharynx, may play a critical role [110]. The close anatomical relationship between MCs and the neurovascular bundle in this region raises the possibility that proximal MC activation not only contributes to neuronal sensitization but may also serve as an initiating event for systemic manifestations of anaphylaxis.

**FIGURE 1:**
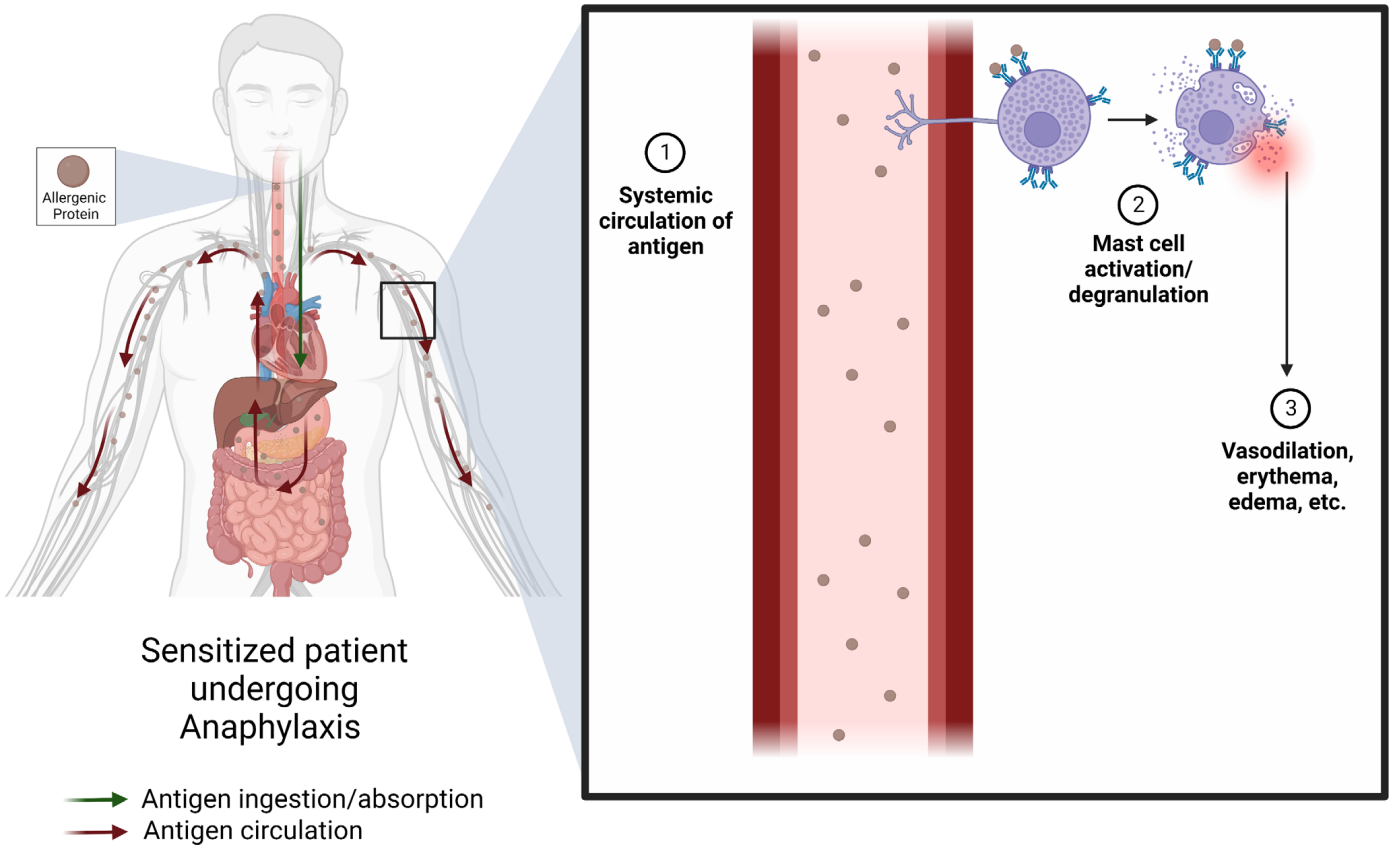
Systemic circulation of antigen in IgE‐mediated food anaphylaxis. Orally consumed allergen is subject to digestion and traversal of the gastrointestinal tract, entering the bloodstream. Mast cell processes detect antigen, resulting in mast cell activation, degranulation, and systemic changes.

Recent studies focused on neuronal modulation of itch in atopic disease have suggested alternative pathways involved in MC signaling [111]. These studies are especially important in understanding the systemic degranulation of MCs and provide unique insights into anaphylactic mechanisms, shedding light on the newfound importance of neuronal signaling in eliciting systemic symptoms. We suggest the dual importance of the systemic presence and distribution of allergen and neuronal MC signaling in IgE‐mediated anaphylaxis (Figures 1 and 3).

## 2 Systemic Antigen in Anaphylaxis

### 2.1 Food‐Induced Anaphylaxis

Food‐induced anaphylaxis presents a prototypical IgE‐mediated hypersensitivity reaction in which ingested allergens may survive digestion, enter the systemic circulation, and trigger widespread MC activation. The systemic presence of antigen is a key determinant in this process, as suggested by studies linking digestive stability to allergenicity [112, 113].

Proteins that retain IgE‐binding epitopes after being subject to digestive processing (pH, temperature, and proteolytic enzymatic alteration) are more likely to cross the gut barrier, enter systemic circulation, and eventually elicit FcεRI crosslinking on MCs [112, 113, 114]. Antigens present conformational (tertiary or quaternary‐folded) and linear (continuous amino acid sequences) epitopes dictating available high‐affinity IgE binding sites, and hence, allergenicity [115]. Both clinical and experimental data support this concept: impaired digestion, as observed in gastric bypass patients and in those on acid‐suppressive therapy, is linked to augmented allergic responses characterized by higher sIgE levels and increased Th2‐cell activation [116, 117, 118]. Conformational epitopes tend not to cause systemic symptoms, as their structures are typically denatured by digestion or heat. Such conformational epitopes account for oral allergy syndrome, whereby many aeroallergens (e.g., pollens, dander, dust mite) cause local symptoms of histamine release in the oropharynx but do not cause systemic symptoms after digestion in the GI tract [115, 119]. Conversely, the potent epitopes of Ara h proteins, the major allergenic proteins of peanuts, are Ara h 2 and Ara h 6; they are recalcitrant to pepsin degradation, efficiently induce basophil degranulation in vitro, and are clinically predictive of true allergy to peanut [120]. Such epitopes are described as sequential/linear and persist despite denaturation. In contrast, Ara h 1 and Ara h 3, though known to be less prevalent peanut allergens, were able to elicit allergic responses after digestive processing. Smits et al. [120] reconciled this finding by hypothesizing a conformational change elicited post‐digestion, allowing for higher affinity IgE binding of Ara h 1 and 3. Antigenic characteristics are also evidenced to impact immune response to allergens. For instance, cow's milk fractions include casein, which is typically present in micellar complexes, and whey lactoglobulin, which is water‐soluble. Accordingly, casein suspended in micellar complexes is predominantly presented to the immune system by Peyer's patches, while whey proteins are highly soluble and rapidly transported across the intestinal epithelium. Activity in the Peyer's patches makes casein a more potent inducer of antibody responses, but whey can induce stronger systemic allergic reactions on exposure, most likely because of its rapid absorption [121]. Together, these findings highlight the complex interplay of digestion and the local immune response in the gut epithelium in acting as the first barrier/modulator for allergen entry into the tissue and bloodstream [120, 121].

After digestive processing, allergenic molecules are proposed to cross the intestinal epithelial barrier in a paracellular or transcellular manner. Paracellular transport, which moves substances between cells, is favored in the context of chronic inflammation, as the overall permeability of the membrane is increased [122, 123]. This allows for a greater amount of allergen to enter Peyer's patches, lamina propria, and the bloodstream, therefore increasing the likelihood of sensitization and hypersensitivity reactions [121, 122, 124]. Studies have also shown that allergen transport in sensitized individuals is primarily facilitated in a transcellular manner through endocytosis, favoring transport of small, hydrophilic proteins [124, 125]. Ara h 2 is well documented to be small and compact, aided by multiple of the protein's disulfide bonds; this is a proposed mechanism of its potent ability to cause hypersensitivity reactions [126, 127].

Two recent studies elaborated on transcellular transport of antigen in the gut endothelia, further modulated by cysteinyl leukotrienes (CysLT) [128, 129]. In sensitized‐tolerant murine models, reduced expression of the CysLT‐metabolizing enzyme dipeptidase 1 led to accumulation of leukotrienes C4/D4, which bound CysLT receptors 1/2 and promoted peanut allergen transport through goblet cells into the bloodstream, activating MCs and triggering anaphylaxis. Confirmation of this relation was achieved with attenuation of anaphylaxis after oral food challenge following administration of zileuton, an inhibitor of CysLT production via arachidonate 5‐lipoxygenase (ALOX5) antagonism; there was no attenuation of anaphylaxis in systemically administered antigen, highlighting the role of CysLT‐facilitated gut transport [128]. Bachtel et al. [129] described similar findings and denoted an additional role of CysLT receptors 1/2 signaling in initiating associated clonal mucosal MC expansion, native to the gut, irrespective of oral anaphylaxis. Gut leakiness has been well documented as both a risk factor and sequela for anaphylaxis with multiple modulators at play; further studies are required to translate these findings from murine to human models [123]. Ultimately, an allergen's capacity to resist digestion, preserve or adopt conformations that enhance IgE binding, and efficiently cross the gut epithelium to reach the bloodstream and lymphoid tissues likely dictates anaphylaxis in IgE‐mediated food allergy (Figure 1).

Additionally, studies have examined the localization of allergens to understand how allergens bound to IgE are acquired by MCs in vivo in mice. Cheng et al. [130] describe the preference of skin MCs to localize perivascularly, and how circulating IgE is captured by the trans‐endothelial cell processes of MCs, thereby sensitizing MCs for allergen‐induced degranulation. This emphasizes the possible importance of a more active mechanism of IgE uptake via the aforementioned cell processes by skin MCs. Though antigen or antigen‐bound IgE are not described as being captured in this manner, further studies must be done to confirm this possibility. Overall, this suggests a greater relevance of IgE binding, and possibly availability, in the bloodstream for MC activation, at least, in producing the cutaneous symptoms of anaphylaxis. Of note, Choi et al. [131] describe perivascularly located dendritic cells communicating with MCs via sampling and transport of allergen via microvesicles, which may bind surface IgE to degranulate MCs. This supports the notion that antigen, present in the systemic circulation via microvesicles, may reach MCs diffusely to induce degranulation, as opposed to a small subset of MCs being activated proximally. Directly contributing to this idea, Strait and colleagues' [132] work demonstrates that food‐induced IgE‐mediated anaphylaxis is attenuated with the administration of serum antigen‐specific IgA and IgG in murine models. They also showed that enteric IgA was not able to suppress anaphylaxis or MC degranulation, suggesting a greater role of systemic antigen distribution in causing anaphylaxis [132].

### 2.2 Alpha‐Gal Syndrome

**FIGURE 2:**
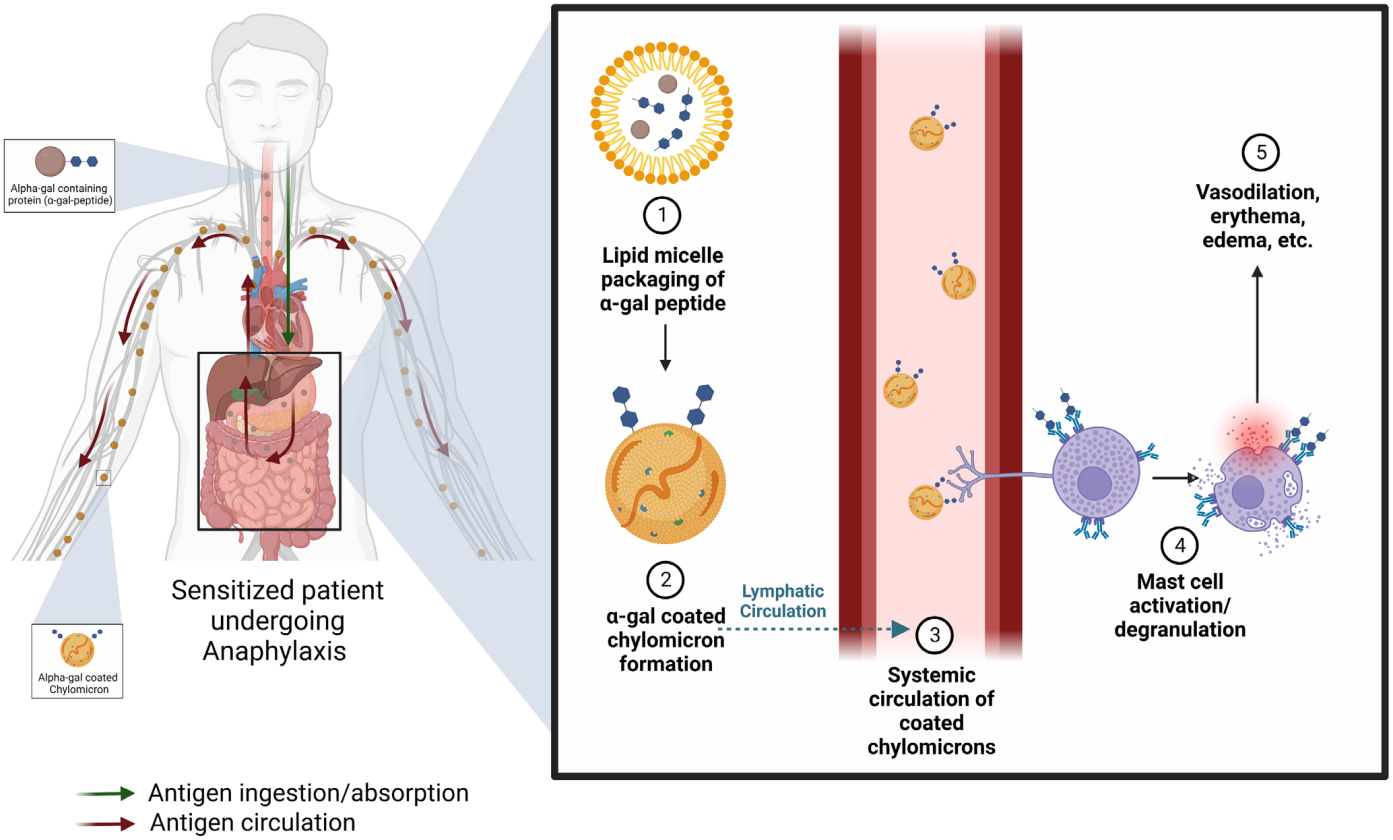
Alpha‐gal syndrome, and chylomicron‐mediated transport resulting in delayed hypersensitivity. Alpha‐gal peptides processed in the gastrointestinal tract are mounted onto the surface of chylomicrons, enter lymphatic circulation, and are detected by the cell processes of perivascular mast cells, triggering mast cell degranulation and delayed, sustained systemic changes.

Pertinent support for the systemic distribution of antigen in IgE‐mediated anaphylaxis is in the unusual case of alpha‐gal syndrome (AGS). This hypersensitivity is to the carbohydrate galactose‐α‐1,3‐galactose (alpha‐gal), present in the monoclonal antibody cetuximab and, naturally, in the tissues of non‐primate mammals: cows, pigs, and sheep [133, 134, 135]. Sensitization occurs via administration of the drug in the case of cetuximab, but more commonly, through repeated Lone Star tick bites [135]. AGS is also unique because of its delayed symptoms, occurring 3–6 h from exposure to the antigen [135]. A hypothetical mechanism for the delayed reaction is the packaging, or masking, of the alpha‐gal antigen conjugated with glycolipids into micelles after digestive processing, which is then incorporated onto the surface of chylomicrons. These chylomicrons then enter (lymphatic) circulation which allows for slow, systemic, antigen binding to IgE on MCs, and consequently, MC degranulation (Figure 2) [133, 136]. The glycolipid hypothesis, termed by Román‐Carrasco et al., suggests that the lipid‐associated digestion of alpha‐gal allows for initial protection from degradation or capture by free or mounted IgE, and that chylomicron‐based transport contributes significantly to the later degranulation of effector cells that result in anaphylaxis (Figure 2) [133, 136]. In vitro studies by Román‐Carrasco et al. [133] examined the ability of alpha‐gal‐containing peptides, extracted from beef and conjugated to lipid, to cross a monolayer of cultured intestinal epithelial cells and, thereafter, degranulate basophils isolated from an alpha‐gal sensitized patient. Results showed that only alpha‐gal conjugated to lipid was able to reach the basolateral side of the epithelium and degranulate basophils, whereas unconjugated alpha‐gal did not cross the epithelium or cause basophilic degranulation [133]. Platts et al. concur with this concept of alpha‐gal glycosylation, and additionally attribute the delayed symptomatology to the slow, systematic dietary processing of these glycolipids. Glycolipids are sequentially processed, over a period of 2–6 h, from large chylomicrons to very low‐density lipoproteins (VLDL) to finally low‐density lipoproteins (LDL), where they become small enough to traverse endothelial walls [135]. Alpha‐gal is able to be glycosylated to LDL; this new moiety would allow for greater LDL penetration and persistence in tissues, hypothetically conferring increased cardiac and allergic risk. With this, in sensitized individuals, alpha‐gal‐conjugated LDL is able to enter the circulation, reach distal tissues, and activate MCs in a characteristically delayed fashion [135]. These studies provide a good model for understanding the infiltration and masking of antigen into the bloodstream, and its presence allows for effector cell degranulation after crossing the initial intestinal barrier (Figure 2).

Further studies may investigate this mechanism to understand how other dietary antigens are affected by the presence of lipids and are spread in circulation in the context of delayed anaphylactic reactions. A key limitation is that most supporting studies have been conducted in vitro and in mice; further studies investigating concurrent lipid and antigen processing, as well as chylomicron formation, in humans and in real time are necessary to confirm the translation of these findings.

### 2.3 Chylomicron Transport of Dietary Antigen

Recent work has highlighted an emerging role for lipid‐associated transport pathways in shaping the allergenicity of dietary allergens. Wang et al. showed that dietary proteins may be facilitated in generating allergic reactions through packaging and systemic distribution in chylomicrons, mediated by long‐chain triglycerides (LCTs) in mice. In the study, ovalbumin (OVA) had increased absorption in the blood via chylomicron‐mediated transport from the intestinal epithelium into mesenteric lymph nodes [137]. The formation of chylomicrons from LCTs was corroborated by the attenuation of antigen absorption with the administration of a chylomicron formation inhibitor. Analysis of mouse plasma via immunoprecipitation also confirmed the packaging of dietary OVA in chylomicrons after 1 h. Also, increased secretion of OVA, associated with chylomicrons, from the CaCo‐2 intestinal epithelial cells was observed [137]. Association between the formation of chylomicrons and OVA secretion was established again with immunoprecipitation, solidifying the coordinated transport of these molecules across the intestinal epithelium. This chylomicron‐dependent mechanism of antigen transport supports the idea that the systemic presence of antigen may be a major contributor to dietary causes of anaphylaxis.

In seeming contrast to this, Li's [138] group examined the effects of dietary medium‐chain triglycerides (MCTs) and LCTs on the absorption and distribution of allergens, and the promotion, overall, of hypersensitivity responses. Mice were gavaged with peanut protein extract delivered in MCTs, LCTs, and LCTs with an inhibitor of chylomicron formation [138]. MCTs were concluded to promote allergic sensitization, as compared to LCTs, and chylomicrons were found to be protective against sensitization and IgE‐mediated anaphylaxis. This is via the proposed mechanism that LCTs efficiently sequester antigen in chylomicrons while in the bloodstream, preventing MC degranulation, while MCTs can bypass the lymphatic system, where transported antigen can degranulate MCs [138]. Both studies emphasize the importance of the systemic presence of antigen, with chylomicron‐ and lipid‐mediated transport likely shaping the nuances of allergen sensitization and reaction elicitation.

Taken together, these findings highlight that systemic availability of dietary antigen—whether enhanced or limited by its mode of lipid transport—plays a critical role in determining sensitization outcomes or anaphylactic potential [137, 138].

### 2.4 Drug‐Induced IgE‐Mediated Anaphylaxis

Drug‐induced anaphylaxis provides another instance of circulatory antigen resulting in systemic degranulation of effector cells. The most common causes of IgE‐mediated anaphylaxis are beta‐lactam antibiotics, cephalosporins, and, less frequently, fluoroquinolones [139, 140]. Severe beta‐lactam‐induced anaphylaxis most often follows parenteral administration, particularly intravenous or intramuscular injection [139, 141, 142]. This suggests that drugs that rapidly reach high concentrations in the bloodstream, remain in circulation and avoid prompt diffusion out of the vasculature, and avoid enteric processing are associated with an increased risk of IgE‐mediated anaphylaxis [139, 141, 143, 144]. In the vasculature, penicillin undergoes haptenation to form hapten‐carrier complexes, which, once circulating, engage antigen‐sIgE and initiate MC degranulation [144, 145]. The intravascular presence of drugs and the capacity to form hapten‐carrier complexes together emphasize a central role of circulating antigen in anaphylaxis. While drug‐induced reactions may also arise through IgE‐independent mechanisms, the findings described above reinforce the concept that systemic allergen availability is critical for IgE‐mediated anaphylaxis [141, 144, 146].

## 3 Neuronal Involvement in Anaphylaxis

Early evidence for nervous system involvement in anaphylaxis originated from a classic study demonstrating Pavlovian conditioning of anaphylactic responses in a murine model [147]. In this experiment, antigen exposure paired with audiovisual cues led to a conditioned response in which audiovisual stimulation alone subsequently induced elevations in serum protease levels, indicative of anaphylaxis. The finding was important in suggesting a more nuanced neuro‐psycho‐immunologic interplay in anaphylaxis [147, 148].

A neuronal component to MC degranulation has been proposed in recent years, suggesting that not all MCs “view” antigen, but receive signaling originating from a subset of distal MCs, and engage in crosstalk with sensory neurons‐ best investigated in studies of itch, nociception, and conditions of chronic inflammation.

### 3.1 Bidirectional Signaling and the Neuro‐Immune Synapse

The close physical proximity of sensory nerve terminals and MCs at the host‐environment interface provides an anatomical basis for bidirectional communication: MCs can activate sensory neurons, while neuronal signals can, in turn, modulate MC activity and vascular responses [90, 149]. Specialized neuro‐immune synapses have been described at these interfaces, where neurotransmitters, neuropeptides, and immune mediators are exchanged to coordinate inflammatory and sensory signaling [150, 151, 152]. In itch, MCs are hypothesized to interact with adjacent cells using pseudopodia‐like projections, degranulating in a piecemeal fashion to selectively release mediators that may contribute to neuronal regulation and pain signaling [66, 153, 154, 155]. This results in key neuromodulation, illustrating MCs' dual proposed function in causing inflammation and potentiating inflammatory signaling [66, 153, 154, 155].

**FIGURE 3:**
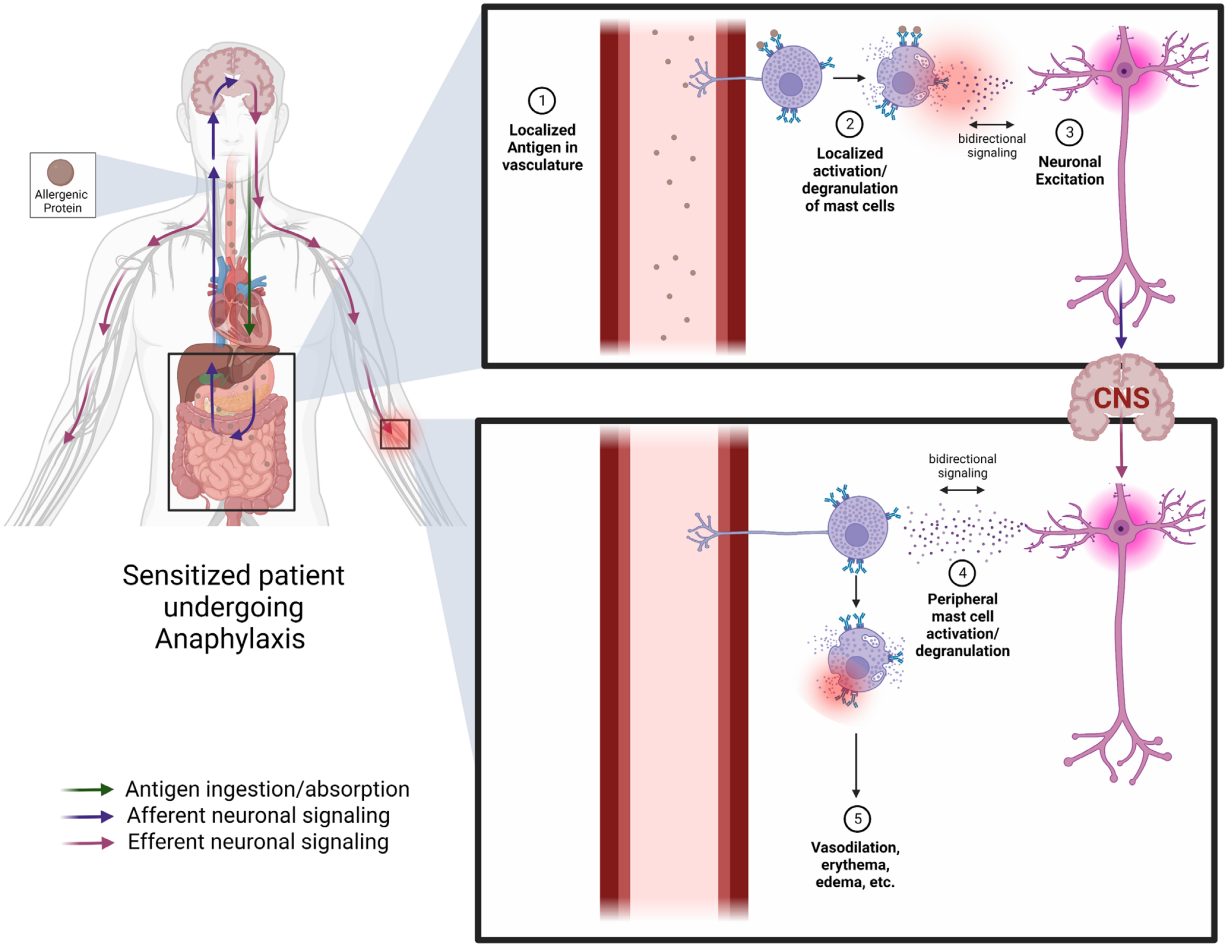
Neuronal signaling in IgE‐mediated anaphylaxis. Perivascular mast cells detect antigen, causing local degranulation. Inflammatory mediator release produces bidirectional signaling between mast cells and neurons, potentiating signaling to the CNS, triggering downstream activation of distal mast cells, eliciting systemic changes.

Studies examining pain signaling and itch outline the importance of histamine and substance P, released from preformed granules in MCs. Receptors for histamine (H1R, H2R, H3R, H4R) are present on neuronal and glial cells [151, 154, 156, 157]. Histamine is a pruritogen, and binding of histamine through its receptors on neural fibers causes the opening of the transient receptor potential (TRP) channel TRPV1/4, resulting in neuronal excitation and itch [151, 154, 156, 157]. Serotonin, another mediator released by MCs, at an even lower dose threshold, is proposed to have similar functions and neuromodulatory effects as histamine [158]. Histamine is vital for homeostasis, particularly in the hypothalamic regulation of arousal, sleep, and feeding. Studies have demonstrated that the release of histamine from the tuberomammillary nucleus and MCs in the brain can modulate the release of, and be modulated by, a variety of different neurotransmitters [159, 160, 161, 162]. MCs are present in the fetal brain but decline in frequency after birth. In adults, histamine is produced primarily by neurons of the tuberomammillary nucleus [157, 163]. Interestingly, in sensitized mouse models allergic to bovine whey, brain MC burden and histamine levels were increased, accompanied by reduced mobility and depression‐like behavior, potentially linked to cortical demyelination [164]. Overall, histamine exerts bidirectional control over neurotransmitter release and signaling, with reciprocal feedback from these neurotransmitters further shaping histaminergic neuromodulation (Figure 3) [149, 162].

Substance P is a neuropeptide released by neural and glial cells that binds to the neurokinin 1 receptor (NK1R). Substance P has been shown to activate MCs and neurons, as well as increase the priming of both, resulting in a lower threshold required for cell activation in the context of itch [165, 166, 167]. Substance P has an associated role in potentiating and increasing inflammatory reactions, illustrating its multifaceted function [148, 168, 169]. Substance P additionally increases gene expression of TNF‐α, possibly potentiating the pro‐inflammatory signaling that occurs between MCs and neurons [148, 170]. In a passive cutaneous anaphylaxis model, studies show that antagonism of substance P and calcitonin gene‐related peptide (CGRP) reduces cutaneous changes post MC degranulation in mice, with even greater attenuation with surgical denervation of peripheral sensory nerves. Siebenhaar et al. [171] hypothesize that these neuropeptides may be involved in this cutaneous anaphylaxis model by increasing MC releasability, degranulation, or by eliciting local hemodynamic effects. Together, these findings lend support to the involvement of these mediators in bidirectional crosstalk between cutaneous MCs and sensory neurons during allergic skin inflammation, which may in turn contribute to cutaneous aspects of the multisystem effects observed in anaphylaxis [148, 171].

Furthermore, protein‐activated receptors (PARs) are a principal group of receptors located on sensory neurons and MCs and are activated by MC‐derived proteases [148, 172, 173, 174, 175]. Studies have investigated the role of PARs in the context of chronic inflammation and pain production. PAR‐2 receptors were found to be upregulated in the keratinocytes of atopic dermatitis patients, suggesting a role in itch and the pro‐inflammatory cascade present in this disease [172, 176]. Zhang and colleagues [177] show that activation of PAR‐2 receptors on microglial cells stimulates the release of TNF and IL‐6, which in turn allows for greater surface receptor expression on microglial cells and MC activation. TNF‐α is also present within preformed granules of MCs, and on release (via an IgE‐dependent mechanism) can bind to tumor necrosis factor receptor‐1 (TNFR1) present on neurons [178, 179]. Interestingly, PARs may be involved in MC‐independent immune sensing by neurons, specifically nociceptors. The mechanism by which PARs detect and potentiate signaling after recognizing allergens is unclear, but it suggests complex neuronal mechanisms by which they act in type 2 inflammation [180, 181, 182, 183].

Tryptase, a key protease, activates the PAR‐2 receptor; it is continuously secreted by MCs, and levels of alpha and beta tryptases have been used as a biomarker for anaphylaxis or increased overall presence of MCs [3, 184]. Tominaga and Fujikawa [185] demonstrate the ability of MCs to communicate with and alter the extent of signaling via sensitization of enteric glial cells via tryptase and PAR‐2 receptors in the context of intestinal inflammation. Though many of the downstream targets of these neuromodulators and overall pathways are disease‐specific and do not necessarily involve the presence of IgE, the proposed mechanisms, which suggest a “positive feedback loop” generated from bidirectional signaling, provide insight into the mechanisms that may contribute to IgE‐mediated anaphylaxis [186]. Numerous mechanisms and mediators are involved in MC activation and neural communication; while this paper will not contain an exhaustive list, the highlighted mediators are but a select key examples supporting neuronal involvement in anaphylaxis. This concept of bidirectional signaling (Figure 3) is of importance and provides a basis for the hypothesis that neuronal signaling may be a contributor to widespread MC activation in anaphylaxis.

### 3.2 Anaphylactic Symptomatology and Neuronal Signaling

A link between histamine and neuronal activation in anaphylaxis is supported by multiple lines of evidence. Histamine has been shown to act on primary afferent neurons, potentiating itch and contributing to immune defense via the release of neurotransmitters from peripheral neurons to signal to the central nervous system (CNS) [187, 188, 189]. In addition, peripheral neurotransmitter release has been shown to enhance histaminergic discharge from adjacent MCs, suggesting a feed‐forward amplification mechanism capable of escalating a modest allergic reaction into anaphylaxis [190].

Supporting a role for neurons in regulating anaphylaxis, work from Rogoz et al. [191] demonstrated that metabotropic glutamate receptor 7 (mGluR7), expressed on peripheral histamine‐sensing neurons, serves as a dampening pathway for histamine‐mediated hypothermia, pruritis, and stereotactic behavior in mice. Intradermal administration of histamine resulted in more severe anaphylactic symptoms when mGluR7 was genetically ablated. Loss of mGluR7 in this model also led to increased levels of plasma histamine and protease‐1 levels compared to wild‐type controls, correlating with a more severe anaphylaxis phenotype [191]. In addition, in mice lacking mGluR7, neurons exhibited increased expression of substance P. MCs express the receptor for substance P, NK1R, and substance P has been shown to promote histamine release from MCs [191, 192]. The results demonstrate that neuronal circuits exert negative feedback control over systemic anaphylaxis symptoms, specifically through an mGluR7‐dependent, MC‐derived histamine‐modulated pathway, and provide mechanistic insights linking neurotransmitter release (substance P) with increased release of histamine from MCs [191]. Thus, these findings provide evidence of neuronal feedback control of systemic anaphylaxis, specifically through a MC‐derived histamine‐modulated pathway (Figure 3).

Additionally, a gut‐brain link in anaphylaxis has been demonstrated experimentally in a murine model of intestinal anaphylaxis. Kreis et al. [193] examined CNS activation, measured by c‐Fos protein expression, a marker of neuronal activity, in a model using intraperitoneal sensitization with OVA and adjuvant followed by direct intestinal challenge, and observed increased activation in the brainstem. This activation was dependent on histamine and serotonin, as pharmacologic antagonists of receptors for these mediators resulted in the abrogation of c‐Fos expression in the brainstem [193].

Furthermore, a neuro‐immune axis has been implicated in allergen‐induced avoidance behavior [194]. In a subcutaneous sensitization model using OVA coupled with alum as an adjuvant, followed by oral administration of OVA, avoidance behavior was analyzed by quantifying licks upon sensitization. Sensitized mice had reduced preference for water containing OVA, comprising avoidance behavior. Findings showed that the avoidance response was dependent on MC sensing of allergen via IgE [194]. Avoidance in this model also corresponded to activation of brain regions canonically involved in promoting aversive responses, including the amygdala, nucleus of the solitary tract, and external lateral parabronchial nucleus [194]. A key role for the MC‐derived mediator, leukotriene C4 (LTC4), was shown in this model. Blockade of the enzyme required for generating LTC4, leukotriene C4 synthase (CTC4S), and genetic ablation of a receptor for LTC4, cysteinyl leukotriene receptor 2 (CysLTR2), resulted in diminished avoidance behavior [194]. Colonic epithelial cell production of growth and differentiation factor 15 (GDF15), which activates nausea‐associated circuits in the area postrema of the brain, was also identified in sensitized mice and found to be dependent upon MCs and IgE [194]. Administration of GDF15 in MC‐deficient mice restored avoidance behavior, indicating that GDF15 acts downstream of MCs. Together, these findings linked peripheral immune sensing of food allergens via MCs and IgE to cellular production of GDF15 in the GI tract, which induces nausea, overall providing a mechanistic framework for how the CNS limits ingestion of potentially harmful allergens. The specific neuroimmune circuitry underlying this behavior remains to be elucidated; vagal ablation failed to modulate avoidance behavior in this work [194]. Additional work dissecting the nociceptive and afferent circuits that link peripheral allergen sensing to centrally mediated avoidance behavior will be important for clarifying the underlying neuroimmune mechanisms. In all, these studies strongly support CNS in addition to peripheral involvement in phenotypes of anaphylaxis.

Lastly, a study by Bao et al. [74] offered more direct evidence that a MC‐neuron network drives the rapid hypothermic response characteristic of anaphylaxis in a passive intravenous 2, 4, 6‐trinitrophenyl (TNP)‐OVA sensitization model with intravenous challenge in mice. Using a chemogenetic TRAP approach, neurons that were naturally activated during anaphylaxis were genetically targeted to express a Gq‐coupled DREADD receptor [74]. Gq‐DREADD is a modified receptor that does not respond to natural ligands but is selectively activated by the drug clozapine. Subsequent activation of these “TRAP‐ed” neurons with clozapine was sufficient to reproduce the anaphylaxis‐associated hypothermic responses, demonstrating that reactivation of anaphylaxis‐engaged neural circuits can drive key features of the reaction. Circuit mapping localized these neurons to established thermoregulatory hubs, particularly the dorsal subnucleus of the lateral parabrachial nucleus and the median preoptic area, which receives afferent input from the lateral parabrachial nucleus [74]. This pathway is known to elicit hypothermia through attenuation of brown adipose tissue (BAT) thermogenesis and promotion of cutaneous vasodilation, which was corroborated in anaphylactic mice [74, 195]. This pathway was further shown to depend on TRPV1‐expressing peripheral sensory neurons. Rescue of TRPV1 selectively in sensory neurons partially restored hypothermia in TRPV1‐deficient mice, and chemogenetic activation of TRPV1^+^ neurons reproduced the hypothermic phenotype. Hypotension was also elicited on activation of TRPV1^+^ sensory neurons. Of note, the authors observed deposition of MC granules along nerve fibers in the ear and trachea by microscopy, suggesting physical contact between released MC granules and sensory neurons of anaphylactic mice, and consequently, that MC granules may directly activate TRPV1^+^ sensory neurons [74]. In support of this, in vitro experiments with cultured primary dorsal root ganglion (DRG) neurons showed calcium influx when exposed to MC granules, indicative of neuronal activation. Bao et al. then examined the role of the MC mediator chymase, predominantly expressed by CTMCs, and found attenuation of hypothermia in mice lacking the subset of CTMCs. Correspondingly, they demonstrated the expression of the chymase receptor PAR1 on TRPV1^+^ DRG neurons. Physiological concentrations of chymase were sufficient to activate TRPV1^+^ neurons in vitro, and this could be blocked by pharmacologic inhibition of chymase. These results demonstrate robust evidence for a MC‐TRPV1 sensory neuron‐central thermoregulatory axis in the induction of hypothermia in anaphylaxis [74]. However, whether similar neuronal mechanisms are engaged during anaphylaxis triggered by direct oral challenge remains to be determined, and the route of antigen exposure may result in differing neurological pathways being invoked. Of note, Bao and colleagues postulate that hypotension may occur via a similar mechanism of action as hypothermia, but they were unable to separately establish this association. Collectively, these results provide growing support for complex MC‐neuronal bidirectional signaling, potentiated with CNS involvement, in producing systemic manifestations of IgE‐mediated anaphylaxis (Figure 3).

## 4 Concluding Remarks

The fundamental mechanisms of IgE‐mediated anaphylaxis have yet to be fully elucidated. Two hypotheses of interest are (1) systemic distribution of antigen contributes to widespread MC degranulation, and (2) activation of a subset of MCs results in neuronal signaling that mediates widespread anaphylactic symptoms, possibly through systemic activation of MCs. The latter hypothesis does not require that all MCs “view” antigen, or that high‐affinity IgE cross‐links a large number of distant MCs for activation and degranulation. There is support for both hypotheses, and emerging evidence highlights a role for additional neuronal signaling, which may account for the very rapid induction of anaphylaxis even with small amounts of allergen, and prior to systemic absorption. We posit a role for both hypotheses, which may differ in involvement depending on specific symptom production and the nature of the implicated agent. The means by which an antigen may gain access to the bloodstream and be processed prior to encountering MCs is paramount in understanding allergenicity and MC activation. Antigens that are naturally processed by the digestive system and systemically distributed, such as food antigens, point to a role for systemic dissemination potentiating anaphylaxis. The rapid induction of widespread phenotypic manifestations of anaphylaxis, such as homeostatic dysregulation and cutaneous changes, may be underpinned by neuronal signaling rather than systemic antigen, as supported by current studies investigating itch and anaphylactic shock.

Further investigation is crucial to develop therapies for genetic conditions, such as mastocytosis, mast cell activation syndrome, and HαT, that increase anaphylaxis risk and severity [196, 197]. Inflammatory mediators and receptors must be outlined, such that targeted therapeutics may be developed. While avoiding a known triggering allergen is effective, prevention remains elusive when the antigen is unknown, such as in idiopathic anaphylaxis. This condition necessitates further understanding of the propagation of anaphylactic symptoms and mechanism‐specific therapies [198]. Moreover, the route of administration of developing therapies may be informed by the nature of the target, but also by the mechanism by which anaphylaxis occurs; targeting of localized populations of MCs, neurons, or circulating antigen must be considered. Studies developing antigen‐specific prophylactic therapies must consider parenteral or enteral routes of administration, depending on whether the circulation and persistence of antigen in the bloodstream is the greatest contributor to the production of anaphylaxis [199].

Mechanistic insights into active models of anaphylaxis are incomplete, and ongoing work is needed to further elucidate the specific neuro‐immune regulatory networks that underlie anaphylaxis in vivo. Continued research is needed not only to confirm either hypothesis but to determine the extent to which each mechanism contributes to the production of specific (systemic) anaphylactic symptoms. Obvious limitations exist in studying anaphylaxis in humans, but humanized murine models are optimal, to date, to mechanistically understand this condition. A key limitation of many murine experimental systems is the use of intraperitoneal or intravenous allergen challenge, or intragastric gavage for antigen delivery, all of which bypass proximal gastrointestinal segments that would normally be engaged under physiologic conditions and may modulate anaphylactic pathways. As anaphylaxis encompasses a variety of symptoms and a range of severity, further understanding of the precise underpinnings of each phenotype is of interest. As with Bao et al.'s findings, mechanisms of hypotension and hypothermia may overlap or differ, necessitating discrete delineation of these pathways. Finally, accounting for patient diversity (genetics, socioeconomic background, and environmental factors) is necessary for determining the nature of anaphylaxis at play and in providing precision‐medicine‐driven care [61].

## Funding

National Institutes of Health, Grant Award Numbers R01AI153708 and R01AI151707. Grant Award from the Job Research Foundation. Institutional funding from the Icahn School of Medicine at Mount Sinai.

## Ethics Statement

The authors have nothing to report.

## Consent

The authors have nothing to report.

## Conflicts of Interest

The authors declare no conflicts of interest.

## Data Availability

No datasets were generated or analyzed during this current study.
